# A novel combination treatment for fragile X syndrome predicted using computational methods

**DOI:** 10.1093/braincomms/fcad353

**Published:** 2024-01-15

**Authors:** Wayne Chadwick, Ivan Angulo-Herrera, Patricia Cogram, Robert J M Deacon, Daniel J Mason, David Brown, Ian Roberts, Daniel J O’Donovan, Michael R Tranfaglia, Tim Guilliams, Neil T Thompson

**Affiliations:** Healx Ltd., Cambridge, CB3 0DU, UK; Healx Ltd., Cambridge, CB3 0DU, UK; Department of Genetics, Faculty of Science, Institute of Ecology and Biodiversity (IEB), University of Chile, Santiago 7800024, Chile; Center for Neural Circuit Mapping, UCI, School of Medicine, University of California, Irvine, CA 92617, USA; Department of Genetics, Faculty of Science, Institute of Ecology and Biodiversity (IEB), University of Chile, Santiago 7800024, Chile; Healx Ltd., Cambridge, CB3 0DU, UK; Healx Ltd., Cambridge, CB3 0DU, UK; Healx Ltd., Cambridge, CB3 0DU, UK; Healx Ltd., Cambridge, CB3 0DU, UK; FRAXA Research Foundation, Newburyport, MA 01950, USA; Healx Ltd., Cambridge, CB3 0DU, UK; Healx Ltd., Cambridge, CB3 0DU, UK

**Keywords:** fragile X syndrome, phosphodiesterase, GABA, ibudilast, gaboxadol

## Abstract

Fragile X syndrome is a neurodevelopmental disorder caused by silencing of the fragile X messenger ribonucleotide gene. Patients display a wide spectrum of symptoms ranging from intellectual and learning disabilities to behavioural challenges including autism spectrum disorder. In addition to this, patients also display a diversity of symptoms due to mosaicism. These factors make fragile X syndrome a difficult syndrome to manage and suggest that a single targeted therapeutic approach cannot address all the symptoms. To this end, we utilized Healx’s data-driven drug discovery platform to identify a treatment strategy to address the wide range of diverse symptoms among patients. Computational methods identified the combination of ibudilast and gaboxadol as a treatment for several pathophysiological targets that could potentially reverse multiple symptoms associated with fragile X syndrome. Ibudilast is an approved broad-spectrum phosphodiesterase inhibitor, selective against both phosphodiesterase 4 and phosphodiesterase 10, and has demonstrated to have several beneficial effects in the brain. Gaboxadol is a GABA_A_ receptor agonist, selective against the delta subunit, which has previously displayed encouraging results in a fragile X syndrome clinical trial. Alterations in GABA and cyclic adenosine monophosphate metabolism have long since been associated with the pathophysiology of fragile X syndrome; however, targeting both pathways simultaneously has never been investigated. Both drugs have a good safety and tolerability profile in the clinic making them attractive candidates for repurposing. We set out to explore whether the combination of ibudilast and gaboxadol could demonstrate therapeutic efficacy in a fragile X syndrome mouse model. We found that daily treatment with ibudilast significantly enhanced the ability of fragile X syndrome mice to perform a number of different cognitive assays while gaboxadol treatment improved behaviours such as hyperactivity, aggression, stereotypy and anxiety. Importantly, when ibudilast and gaboxadol were co-administered, the cognitive deficits as well as the aforementioned behaviours were rescued. Moreover, this combination treatment showed no evidence of tolerance, and no adverse effects were reported following chronic dosing. This work demonstrates for the first time that by targeting multiple pathways, with a combination treatment, we were able to rescue more phenotypes in a fragile X syndrome mouse model than either ibudilast or gaboxadol could achieve as monotherapies. This combination treatment approach holds promise for addressing the wide spectrum of diverse symptoms in this heterogeneous patient population and may have therapeutic potential for idiopathic autism.

## Teaser

Chadwick *et al*. utilized computational methods to identify two drugs to treat fragile X syndrome. As monotherapies, one of the drugs improved cognition and the other normalized autism spectrum disorder–like behaviours in a mouse model of fragile X syndrome. Combined treatment with both drugs reversed cognitive and behavioural deficits.

## Graphical Abstract

**Graphical Abstract:**
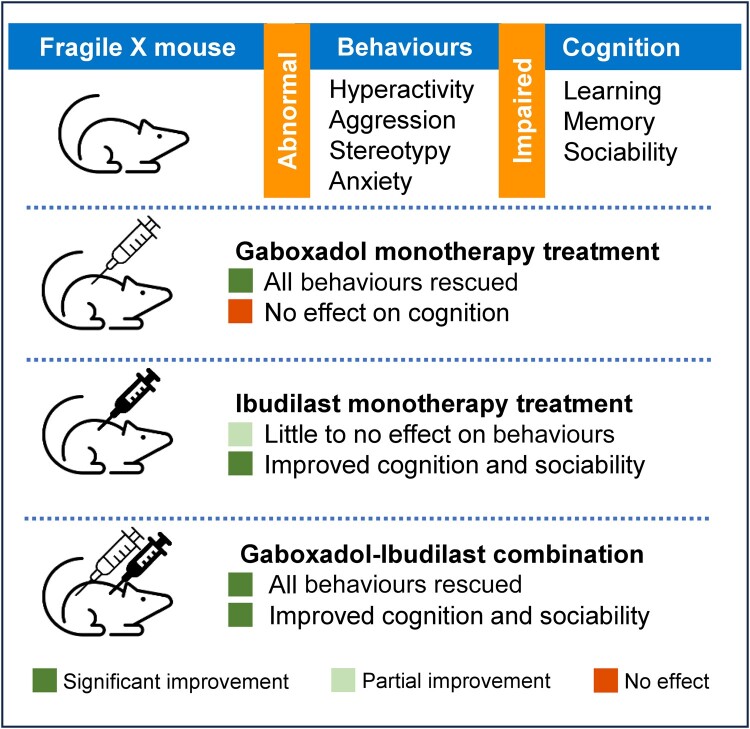

## Introduction

Fragile X syndrome (FXS) is the most commonly inherited form of single-gene mutation that causes a range of developmental problems. FXS is typically characterized by mild to severe cognitive dysfunctions with associated mood, social and behavioural challenges including autism spectrum disorder, attention-deficit hyperactivity disorder and aggression.^1^

FXS is caused by a full mutation of the fragile X messenger ribonucleotide (*FMR1*) gene, which arises from the hypermethylation of a cytosine–guanine–guanine trinucleotide repeat expansion.^2^ A full mutation consists of >200 repeats, resulting in epigenetic silencing of *FMR1* leading to loss of expression.^1^  *FMR1* encodes fragile X messenger ribonucleotide protein (FMRP), an RNA-binding protein involved in the regulation of editing, translation and transporting of neuronal mRNAs.^3^ FMRP associates with thousands of mRNA targets in the brain, which in turn affects a wide range of neuronal processes and functions.^4-6^ Some of these mRNA targets include a large fraction of the pre- and postsynaptic proteome including ion channels which regulate cellular excitability, as well as transcription factors and chromatin-modifying proteins that can affect the genetic and proteomic content of cells. FMRP also interacts directly with both voltage- and ligand-gated ion channels and, in so doing, can manipulate neuronal excitability.^7^

Due to the ubiquitous expression of FMRP and its ability to regulate a large portion of the neuronal proteome, it is not surprising that loss of this protein has far-reaching consequences. The broad spectrum of symptoms associated with FXS, ranging from behaviours to cognition, shows the important role FMRP plays in neuronal development, functioning and network formation. FXS patients also display considerable clinical and genetic heterogeneity, which manifests in a wide spectrum of behavioural phenotypes among patients.^8^ This heterogeneity is thought to stem from the heterogeneous genetic background of patients as well as the existence of mosaicism of *FMR1* methylation, which results in a differential expression of *FMR1* across the brain.^9^ It is therefore not surprising that despite several potential targets being uncovered and trialled in the clinic,^10^ no disease-modifying therapy currently exists that is able to address the multiple symptoms in FXS patients.

It is, therefore, reasonable to suggest that a multitargeted polypharmacological approach would be best suited to address the broad range of symptoms in this heterogeneous patient population. Here, we propose a combination of two drugs, ibudilast and gaboxadol, from different classes for the treatment of FXS. This combination was first identified using Healx’s data-driven drug discovery platform as a candidate that may effectively rescue a complementary set of phenotypes, based upon the combined action against putative targets and other information our platform had gathered about drug and disease. Ibudilast is a broad-spectrum phosphodiesterase (PDE) inhibitor, with a preference for PDE3, PDE4, PDE10 and PDE11^11^ and has shown to have several beneficial effects in the brain.^12-14^ The therapeutic benefits of PDE inhibition in FXS patients were demonstrated with BPN14770, a PDE4D inhibitor, which significantly improved the cognitive performance in this patient population,^15^ who have reduced levels of cyclic adenosine monophosphate (cAMP).^16^ THIP/gaboxadol is a GABA_A_ receptor agonist with a preference for extrasynaptic GABA_A_ receptors containing alpha4beta3 and delta subunits.^17,18^ Loss of GABAergic signalling in FXS results in excitatory and inhibitory imbalance, which contributes to the pathophysiology of FXS.^19^ This mechanism is known as the GABAergic hypothesis, which is supported by the fact that individuals with FXS have reduced GABA_A_ receptor availability^20^ as well as altered GABA transport and synthesis.^21-23^ Gaboxadol was found to be safe and well tolerated in FXS patients following a Phase 2a clinical assessment. In addition to this, gaboxadol also demonstrated an initial efficacy signal based on clinician- and caregiver-rated end-points that assessed behaviours such as hyperactivity, irritability, stereotypy and anxiety.^24^

Here, we demonstrate that monotherapy treatment with ibudilast or gaboxadol was able to rescue distinct phenotypes in *Fmr1* knockout (KO) mice. Gaboxadol was highly efficacious in rescuing behaviours, typically associated with FXS such as aggression, anxiety, hyperactivity and stereotypy, while ibudilast effectively reversed cognitive deficits in *Fmr1* KO mice. Importantly, we demonstrate that ibudilast and gaboxadol co-treatment was able to rescue more phenotypes, including behaviours and cognitive deficits, in *Fmr1* KO mice than either drug could achieve as monotherapies. This polypharmacological approach of targeting multiple pathways linked to FXS pathophysiology could allow for multiple phenotypes to be treated in a clinical population, which displays a wide and diverse spectrum of symptoms.

## Materials and methods

### Computational prediction methods

Given that FXS patients exhibit multiple and diverse symptoms, a drug combination treatment approach is attractive, in particular if the prospective combination is able to address multiple symptoms. However, identifying the most appropriate combination of approved drugs in existence can be challenging. For example, considering a combination of two potential drugs from the ∼4000 that are approved worldwide at a single dose would require ∼8 000 000 laboratory experimental tests, a prohibitively large task if performed manually. Moreover, the majority of combinations would likely be non-efficacious or even toxic. At Healx, we have implemented a suite of combination prediction algorithms that consider multiple data sets and treatment hypotheses to help solve this challenge. The combination of ibudilast and gaboxadol described here was discovered after being predicted by two of our methods that constitute part of the Healx data-driven drug discovery platform: Combination Gene Expression Matching (CGEM) and Target Optimisation (TargOpt; Supplementary Fig. 1A and B).

The CGEM method was based upon a connectivity-mapping scoring system that chooses combinations of drugs based upon the maximal reversal of differentially expressed genes in the disease.^25^ The compound gene expression data were obtained from the CMap LINCS gene expression resource^26^ (https://clue.io/data/CMap2020#LINCS2020; accessed October 30, 2023). The FXS differentially expressed genes used for this analysis were generated from the Gene Expression Omnibus (GEO) data set GSE62721^27^ and prepared as described previously.^28^ The TargOpt method was based upon an algorithm that chooses combinations of drugs based upon the simultaneous maximizing of the number of on-target effects and minimizing of the number of off-target effects for a set of disease-related targets.^29^ The drug and disease targets were obtained via curation and the Healx data-driven drug discovery platform. We coupled the predictions from these approaches with a number of annotations that assist a human expert to prioritize a subset of predictions for experimental validation by taking into consideration the possibility for synergy and adverse drug interactions.

### Animals

*Fmr1* knockout 2 (*Fmr1* KO) mice were generated by deletion of the promoter and first exon of *Fmr1*.^30^ The *Fmr1* KO is both protein and mRNA null. In this study, we used *Fmr1* KO and wild-type (WT) littermates generated on a C57BL/6J background and repeatedly backcrossed onto a C57BL/6J background for more than eight generations.

### Animal housing

The *Fmr1* KO mice were housed in four per cage groups of the same genotype in a temperature- and humidity-controlled room with a 12-h light–dark cycle (lights on 7 a.m.–7 p.m.). Mice were housed in commercial plastic cages (40 × 23 × 12 cm) with Aspen bedding and without environmental enrichment on a ventilated rack system. Food and water were available *ad libitum*, except during test sessions. Testing was conducted during the light phase on male *Fmr1* KO mice and their WT littermates. All experiments were conducted by experimenters who were blind to genotype and drug treatment. Experiments were conducted in line with the requirements of the United Kingdom Animals (Scientific Procedures) Act, 1986. WT animals were assigned to the WT vehicle group, while each KO mouse was assigned to one of the KO treatment groups using randomization. At the end of the study, animals were sacrificed by cervical dislocation.

### Dosing and behavioural assays

*Fmr1* KO and WT littermate mice, whether receiving acute or chronic treatment, were injected intraperitoneal (i.p.) with vehicle [10% DMSO in 90% (20% Captisol in saline)] or gaboxadol or ibudilast or gaboxadol and ibudilast in combination. All drugs were formulated and dosed in the vehicle solution. Administration volumes were 3.85 mL/kg, such that an adult mouse weighing 26 g received a 0.1 mL injection volume. For all test articles, the volume to be administered was based on each mouse’s body weight. Treatment groups remained the same over the course of behavioural testing.

Mice receiving acute treatment were dosed 30 min prior to behaviour testing (refer to Table 1 for dosing regimen). Mice receiving acute dosing were dosed with vehicle on the days they did not receive drug to ensure consistency of stress and handling with the chronically dosed mice. Behaviour tests were separated by a minimum of 3 days, during which the mice receiving acute dosing were dosed with vehicle solution.

**Table 1:** Dosing regimen for acute versus chronic gaboxadol study

Group (*n* = 10)	Drug	Dose (mg/kg)	Drug regimen
WT	Vehicle		QD-2 weeks
*Fmr1* KO	Vehicle		QD-2 weeks
*Fmr1* KO	Gaboxadol	0.15	QD-2 weeks
*Fmr1* KO	Gaboxadol	0.5	QD-2 weeks
*Fmr1* KO	Gaboxadol	1.5	QD-2 weeks
*Fmr1* KO	Gaboxadol	0.15	Acute-30 min
*Fmr1* KO	Gaboxadol	0.5	Acute-30 min
*Fmr1* KO	Gaboxadol	1.5	Acute-30 min

QD, once daily dosing.

For chronic dosing, mice were dosed daily for 2 weeks before any behavioural testing (refer to Table 2 for dosing regimen). For combination dosing, each drug/vehicle was administered separately on either the left or right lower abdominal quadrant via the i.p. route. Following the 2 weeks of pretreatment, all mice continued their dosing regimen, outlined in Table 2, until the completion of all the behavioural phenotyping. On the day of behavioural testing, mice were dosed 30 min before the start of the behavioural assay. All behaviour tests were separated by a minimum of 3 days, during which time dosing continued.

**Table 2:** Dosing regimen for combination study

Genotype (*n* = 10)	Drug 1		Drug 2		Drug regimen
	Name	Dose (mg/kg)	Name	Dose (mg/kg)	
WT	Vehicle		Vehicle		QD
*Fmr1* KO	Vehicle		Vehicle		QD
*Fmr1* KO	Gaboxadol	0.5	Vehicle		QD
*Fmr1* KO	Gaboxadol	1.5	Vehicle		QD
*Fmr1* KO	Vehicle		Ibudilast	6	QD
*Fmr1* KO	Vehicle		Ibudilast	6	BID
*Fmr1* KO	Gaboxadol	0.5	Ibudilast	6	QD
*Fmr1* KO	Gaboxadol	0.5	Ibudilast	6	BID
*Fmr1* KO	Gaboxadol	1.5	Ibudilast	6	QD
*Fmr1* KO	Gaboxadol	1.5	Ibudilast	6	BID

All mice received the same number of daily i.p. injections with either drug or vehicle to maintain consistency of handling. QD, once daily dosing; BID, twice daily dosing (morning and evening).

Each behavioural test was performed between 8 a.m. and 4 p.m. Mice were dosed in the housing room (30 min prior to testing) and then brought to the experimental room to acclimate for 20 min before testing. Animals were tested in only one behavioural task on each experimental day, and each additional behavioural test was separated by at least 3 days. Prior to each test, a mouse that was not included in the study was placed in the experimental apparatus for 3 min. Then, this non-study animal was removed, and the apparatus was cleaned with moist and dry tissues before placing a study mouse into the apparatus. The aim was to create a low but constant background mouse odour for all experimental subjects. Experimenters were blinded to mouse genotype and treatment throughout all behavioural tests and data analysis.

### Open-field hyperactivity

An open-field apparatus was used to test hyperactivity and habituation to a novel environment, in which decreased exploration as a function of repeated exposure to the same environment may be an index of memory. Each mouse was exposed individually to the open field in one session corresponding to 30-min posttest article administration. The open-field assay was performed using an automated system including a Noldus activity monitor chamber with the associated EthoVision software (Noldus Information Technology Inc., Leesburg, VA, USA). A mouse was placed into a corner square facing the wall and horizontal locomotor activity, measured as distance travelled in centimetres (cm) by the number of squares entered with the whole body, was recorded for 30 min.

### Self-grooming (stereotypy)

Stereotypy is measured by an increase in repetitive self-grooming behaviour in the *Fmr1* KO mice. After the 30-min drug pretreatment time elapsed, a mouse was individually placed in an empty VersaMax activity monitor chamber. Following an initial 10-min habituation phase, self-grooming was measured for 10 min using an automated system with the associated VersaDat software (AccuScan Instruments, Columbus, OH, USA).

### Novelty-suppressed feeding or hyponeophagia

The novelty-suppressed feeding test, in which a highly palatable but novel liquid food was available for consumption in a novel environment, measured the latency to consume a defined amount of the novel food as an index of anxiety-like behaviour. Mice were food restricted overnight and tested the next morning. Twenty minutes prior to the test, each mouse was individually placed into a temporary holding cage to prevent social transmission of food preferences. Testing was conducted in a chamber (30 cm length × 30 cm width × 5 cm height) with three white walls and a fourth wall of transparent plastic to allow observation of the mouse. A food well (1.2 cm diameter, 0.9 cm height) was glued to the white Perspex base of the test chamber. An individual mouse was placed into the chamber facing away from the food well containing sweetened condensed milk diluted 50:50 with water. The latency from placement in the test chamber to the start of a proper drinking bout, defined as drinking continuously for 3 s, was measured. Mice that did not drink the novel food during the 5-min test received the maximal latency score.

### Aggression

Offensive aggressive behaviour was measured as the number of mounts or the latency to the first attack of an unfamiliar conspecific. Mounting is a dominance behaviour that consists of attempts to climb on top of another animal. The test chamber was an empty commercial plastic cage (40 × 23 × 12 cm) with a Perspex lid to facilitate viewing of the subjects. An experimental mouse and a novel, WT mouse (with no prior contact with the test mouse) were placed in the cage simultaneously for a 3-min test. The total number of mounts was recorded from above with a light-sensitive video camera using the Noldus EthoVision XT system (Noldus Information Technology Inc., Leesburg, VA, USA).

### Novel object recognition

Recognition memory of a familiar object compared to a novel object was assessed by the novel object recognition (NOR) task. A Plexiglas box (26 cm length × 20 cm width × 16 cm height) and two unique objects (4–6 cm diameter × 2–6 cm height), each in duplicate, were used. Mice were habituated individually to the experimental environment by allowing them to freely explore the box, which was empty, for 20 min per day for two consecutive days before testing. The test involved two consecutive trials, each 5 min in duration. For trial one, two identical objects were placed in the box, and the mouse was allowed to freely explore the objects for 5 min. These objects would be the familiar (f) objects. For trial two, one familiar object (f) is replaced with one novel object (n), and the mouse is allocated 5 min to explore. Object exploration was defined as the mouse sniffing or touching the object with its nose, vibrissa, mouth or forepaws. Time spent near or standing on top of the objects without interacting with the object was not counted as exploration. During the trial, a mouse was required to explore the objects for a minimum of 3 s for that individual animal to be included in the data analysis. For the test trial, the time spent exploring the novel object and the time spent exploring the familiar object were recorded for each mouse. Data were reported as the discrimination index (D2 score). The D2 score was calculated as follows: D2 score = (Time spent exploring novel object − time spent exploring familiar object)/(Total time spent exploring novel and familiar objects).

### Social recognition

In the three-chambered social novelty task, a subject mouse was evaluated for its preference to explore a novel versus a familiar social stimulus mouse, defined as the time spent in the chamber with the novel mouse versus the chamber with the familiar mouse. The apparatus was a rectangular three-chambered box, in which each chamber measured 20 cm (length) × 40.5 cm (width)×22 cm (height). Dividing walls were made from clear Perspex, with openings (10 cm width × 5 cm height) that allowed access into each chamber. The apparatus was lit from below (10 lx).

The test involved three consecutive phases: habituation, sociability and social novelty. During the habituation phase, an individual test mouse was placed in the middle chamber and allowed to freely explore all three chambers, which were empty, for 5 min. Then, the mouse was placed in an opaque holding cage for 3 min, while the apparatus was prepared for the sociability phase. During the sociability phase, the mouse was allowed to freely explore all three chambers, in which one side chamber contained an unfamiliar mouse (stranger one, with no prior contact with the test mouse) and the other side chamber was empty, for 10 min. The stranger mouse was enclosed in a circular wire cage (11 cm in height, bottom diameter of 10.5 cm and bars spaced 1 cm apart; Galaxy Cup, Spectrum Diversified Designs, Inc., Streetsboro, OH, USA), which allowed nose-to-nose contact between the bars. Animals serving as strangers were male mice previously habituated to placement in the cage for 10 min prior to testing. Then, the test mouse was placed in a holding cage for 3 min, while the apparatus was prepared for the social novelty phase. During the social novelty phase, the mouse was allowed to freely explore all three chambers, in which one side chamber still contained a familiar mouse (n) and the other side chamber now contained a novel mouse (n) for 10 min. The novel mouse was enclosed in a wire cage identical to that enclosing the familiar mouse. For each phase of the test, the amount of time spent in each chamber was recorded. An entry was defined as all four paws in one chamber. Data were reported as the discrimination index (D2 score). The D2 score was calculated as follows: D2 score = (Time spent exploring novel mouse − time spent exploring familiar mouse)/(Total time spent exploring novel and familiar mice).

### Object location

The Object–Location Memory task is useful for assessing cognitive deficits in transgenic strains of mice and for evaluating novel chemical entities for their effect on cognition. Testing occurs in an open-field arena, to which the animals are first habituated. The next day, two objects of similar material but different shapes are introduced to the arena. They are spaced roughly equidistant from each other with space in the middle for introducing the subject. In the trial, the animal is allowed to explore the arena with the two objects, and shortly thereafter, the animal again encounters the two objects, except that one of them has switched positions. The trials are recorded using a camera mounted (Noldus) above the arena and scored for the percentage preference for the object in the new location using EthoVision (Noldus). Data were reported as the discrimination index (D2 score). The D2 score was calculated as follows: D2 score = (Time spent exploring novel location − time spent exploring familiar location)/(Total time spent exploring novel and familiar locations).

### Contextual fear conditioning

Contextual fear conditioning (cFC) was performed as previously described.^31^ The mice were placed individually in the contextual chamber (Coulbourn Instruments) and allowed to move freely for 2 min before a mild foot shock (0.7 mA, 2 s) was delivered. The mouse remained in the chamber for 1 min and was returned to its home cage. Twenty-four hours later, the trained mouse was re-introduced to the contextual chamber for 2 min, during which its freezing behaviour (i.e. immobility) was scored.

### Statistical analysis

Data were analysed using GraphPad Prism (GraphPad Software, LLC, version 8.3.0, San Diego, CA, USA). Parametric data were analysed using one-way ANOVA followed by *post hoc* comparisons with Dunnett’s or Sidak’s multiple comparison tests where appropriate. Non-parametric data were analysed using the Kruskal–Wallis one-way ANOVA followed by Dunn’s multiple comparison test. An effect was considered significant if *P* < 0.05.

## Results

### Gaboxadol dose range finding studies

Other groups have reported that a single dose treatment of gaboxadol significantly reversed several phenotypes in preclinical FXS mouse models, including auditory startle response, hyperactivity, stereotypy and aggression.^23,32-34^ This drug class has however previously demonstrated pharmacoresistance and loss of efficacy following chronic dosing in other disorders.^35,36^ In addition to this, several mGluR5-negative allosteric modulators have also shown treatment resistance following chronic dosing in FXS mice. This tolerance is thought to be a potential factor behind their lack of success in the clinic.^37^ To ensure repeated gaboxadol dosing did not affect its potency in FXS mice, we compared the efficacy, through behavioural phenotyping, of acute to chronic dosing. For chronic dosing, mice were dosed for 2 weeks prior to behaviour testing and dosing was maintained until all behaviour experiments were complete. The mice receiving the acute treatment were dosed 30 min prior to any behaviour testing with a 3-day washout between all behaviour assays. Mice receiving acute treatment were dosed with vehicle between washout periods to maintain consistency of handling. Doses were selected based on previously reported efficacy in FXS mouse models for gaboxadol.^33^ Mice received 0.15, 0.5 or 1.5 mg/kg gaboxadol according to Table 1 dosing regimen.

**Figure 1:**
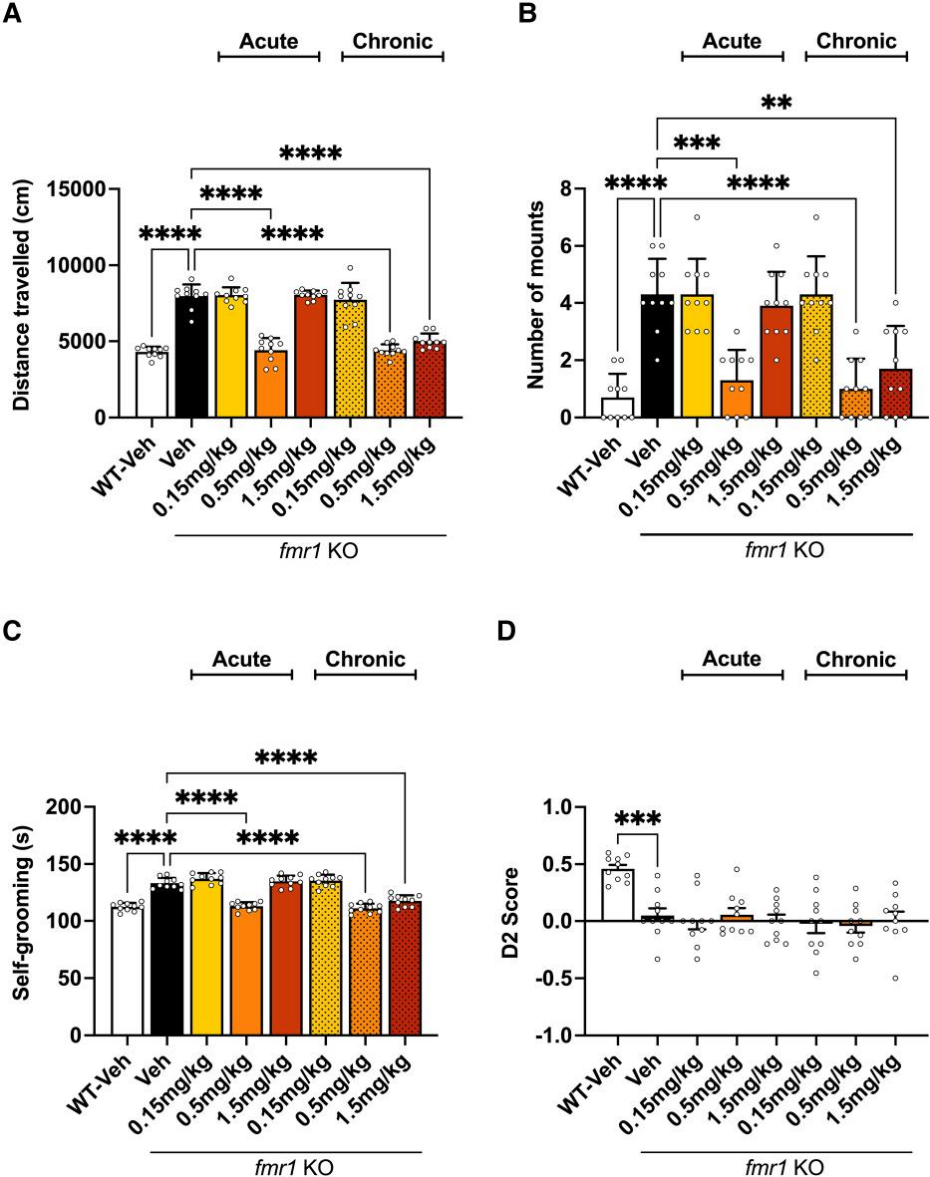
**Comparative effects of acute and chronic dosing of gaboxadol on behaviours in *Fmr1* KO mice.** (**A**) Total distance travelled (cm) by *Fmr1* KO and WT mice in the open field over 30 min. Data analysed by Brown–Forsythe and Welch ANOVA [*F*(7,40.2) = 79.27, *P* < 0.0001] followed by Dunnett’s T3 multiple comparison test. (**B**) Aggression measured by the number of mounts performed by *Fmr1* KO and WT mice. Data analysed by Brown–Forsythe and Welch ANOVA [*F*(7,65.74) = 18.85, *P* < 0.0001] followed by Dunnett’s T3 multiple comparison test. (**C**) Time in seconds (s) *Fmr1* KO and WT mice spent self-grooming. Data analysed by Brown–Forsythe and Welch ANOVA [*F*(7,67.28) = 67.23, *P* < 0.0001] followed by Dunnett’s T3 multiple comparison test. (**D**) NOR D2 score obtained by *Fmr1* KO and WT mice. Data analysed by ordinary one-way ANOVA [*F*(7,72) = 6.748, *P* < 0.0001] followed by Dunnett’s multiple comparison test. Bars indicate mean values (mean ± SEM). Points correspond to values from individual mice. Asterisks represent significant change: ****P < 0.0001, ***P = 0.0001, and **P = 0.039 versus *Fmr1* KO vehicle group. *n* = 10.

Vehicle-treated *Fmr1* KO mice display a hyperactive phenotype by traveling a significantly greater distance in the open field (Fig. 1A) compared to WT mice. Acute and chronic treatment with 0.5 mg/kg gaboxadol significantly reduced locomotor activity back to levels observed in the WT vehicle group (Fig. 1A). Chronic, but not acute, treatment with 1.5 mg/kg gaboxadol was also efficacious in reducing hyperactivity (Fig. 1A). The *Fmr1* KO vehicle–treated mice were significantly more aggressive in comparison to WT vehicle–treated mice, as measured by mounting episodes (Fig. 1B). Mounting is seen as a sign of dominance between two male mice and as such can be interpreted as a sign of aggression.^33^ Acute treatment with 0.5 mg/kg gaboxadol was efficacious in reducing the aggressive phenotype in *Fmr1* KO mice. Chronic doses of 0.5 or 1.5 mg/kg was also efficacious in reducing the aggressive phenotype (Fig. 1B).

The *Fmr1* KO vehicle–treated mice also displayed an increase in stereotypy, assessed by repetitive self-grooming (Fig. 1C). Both acute and chronic doses of 0.5 mg/kg gaboxadol were able to significantly reduce stereotypy (Fig. 1C) in *Fmr1* KO mice back to levels observed in WT vehicle–treated mice. Similarly, chronic 1.5 mg/kg gaboxadol also significantly reduces self-grooming (Fig. 1C) in *Fmr1* KO mice. Cognition was assessed using NOR, which tests an animal’s ability to differentiate between a familiar object and a novel object. If the test animal is able to differentiate between the two objects, it will naturally show a preference for investigating the novel object and, as a result, will have a higher D2 score. The *Fmr1* KO vehicle–treated mice cannot recall interacting with the familiar object, and as a result, the time spent investigating both objects is equivalent (Supplementary Fig. 2), resulting in a low D2 score. Neither acute nor chronic doses of gaboxadol were able to reverse the cognitive deficit in *Fmr1* KO mice for the NOR task (Fig. 1D) or the object location (OL) assay (Supplementary Fig. 3).

The difference in the dose efficacy profile between the acute and chronic treatment regimens is not uncommon for this drug class.^38^ A number of factors are proposed to contribute to altered sensitivity of GABA receptor modulators following chronic dosing, such as altered GABA receptor surface expression, changes in subunit expression, altered receptor coupling and modified intracellular signalling.^39^ Although the exact process driving the efficacy of the chronic 1.5 mg/kg dose warrants further investigation, it does highlight how chronic dosing with this drug class can affect dose selection. This underlines the importance of comparing acute to chronic dosing in a disease-relevant model when performing efficacy-based dose selection studies. Importantly, the acute and chronic 0.5 mg/kg doses showed similar efficacy for all behaviours measured implying there was no loss of potency due to tolerance following chronic dosing of gaboxadol in *Fmr1* KO mice.

As this class of drug has the potential to induce sedation, we needed to ensure that the reduced hyperactivity, stereotypy and aggression in *Fmr1* KO mice, following chronic dosing, were not due to sedative effects. For this experiment, WT mice were chronically dosed with 0.5 or 1.5 mg/kg of gaboxadol for 14 days before subjecting them to an open-field test, to measure activity, and self-grooming, to assess natural behaviour. A vehicle and a 0.5 mg/kg gaboxadol *Fmr1* KO–treated group were included as internal controls to monitor consistency of behavioural end-points and pharmacological efficacy between studies.

**Figure 2:**
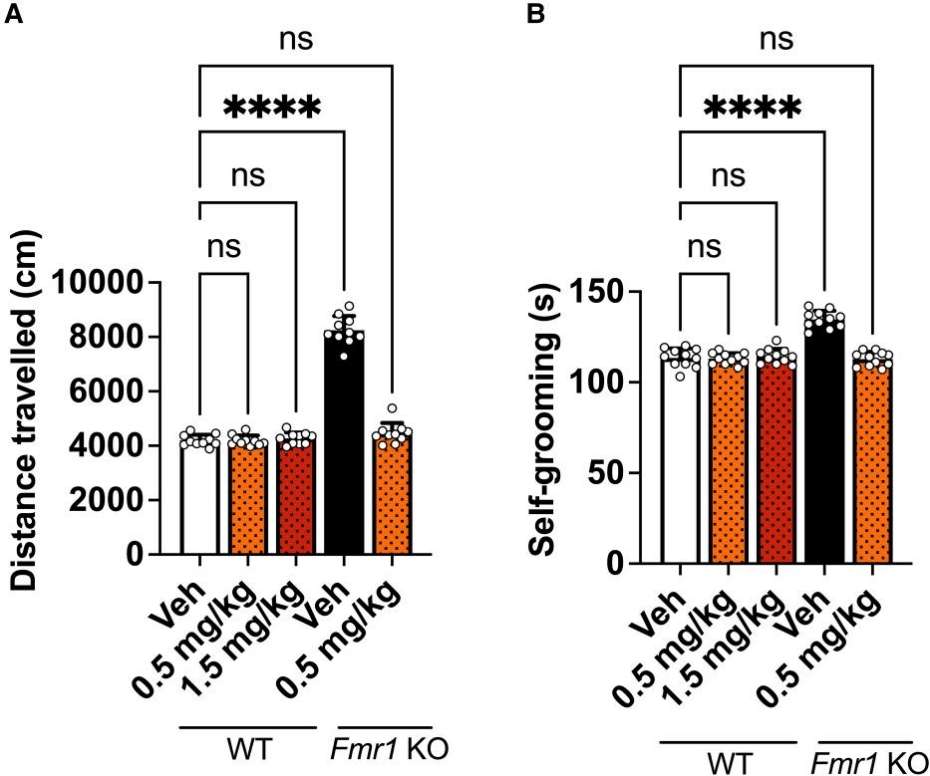
**Effects of chronic gaboxadol dosing in WT and *Fmr1* KO mice.** (**A**) Total distance travelled (cm) by *Fmr1* KO and WT mice in the open field over 30 min. Data analysed by one-way ANOVA [*F*(4,45) = 286.4, *P* < 0.0001] followed by Dunnett’s multiple comparison test. (**B**) Time in seconds (s) *Fmr1* KO and WT mice spent self-grooming. Data analysed by one-way ANOVA [*F*(4,50) = 54.01, *P* < 0.0001] followed by Dunnett’s multiple comparison test. Bars indicate mean values (mean ± SEM). Points correspond to values from individual mice. Asterisks represent significant change; ns, not significant; ****P < 0.0001. *n* = 10.

Consistent with previous studies (Fig. 1A and C), *Fmr1* KO vehicle–treated mice were significantly more hyperactive (Fig. 2A) and showed increased stereotypy (Fig. 2B) compared to WT vehicle–treated mice. As demonstrated previously, 0.5 mg/kg chronically dosed gaboxadol significantly reduced the hyperactivity and stereotypy back to levels observed in the WT vehicle–treated mice. The WT mice dosed with chronic gaboxadol, 0.5 or 1.5 mg/kg, showed no signs of lethargy or reduced activity throughout the 14 days and showed an activity level comparable to vehicle-dosed WT mice on the final day of dosing. Similarly, the grooming behaviour was comparable to the WT vehicle–mice. From this, it can be concluded that the efficacy observed, in the *Fmr1* KO mice, following 0.5 or 1.5 mg/kg chronically dosed gaboxadol, is not a result of sedation. In addition to this, no adverse effects were reported in WT or *Fmr1* KO mice following chronic or acute dosing of gaboxadol.

### Ibudilast dose range finding studies

Both cAMP and cyclic guanosine monophosphate (cGMP) are essential to support long- and short-term memory consolidation, and PDE4 inhibition has been shown to improve learning and memory in FXS patients, by increasing cAMP availability.^15^ Ibudilast is a broad-spectrum PDE inhibitor that allows for the maintenance of both cAMP and cGMP levels. This makes ibudilast a promising potential therapeutic for FXS. To identify the lowest efficacious dose of ibudilast, *Fmr1* KO mice were dosed with 3 or 6 mg/kg ibudilast for 14 days prior to phenotyping.

**Figure 3:**
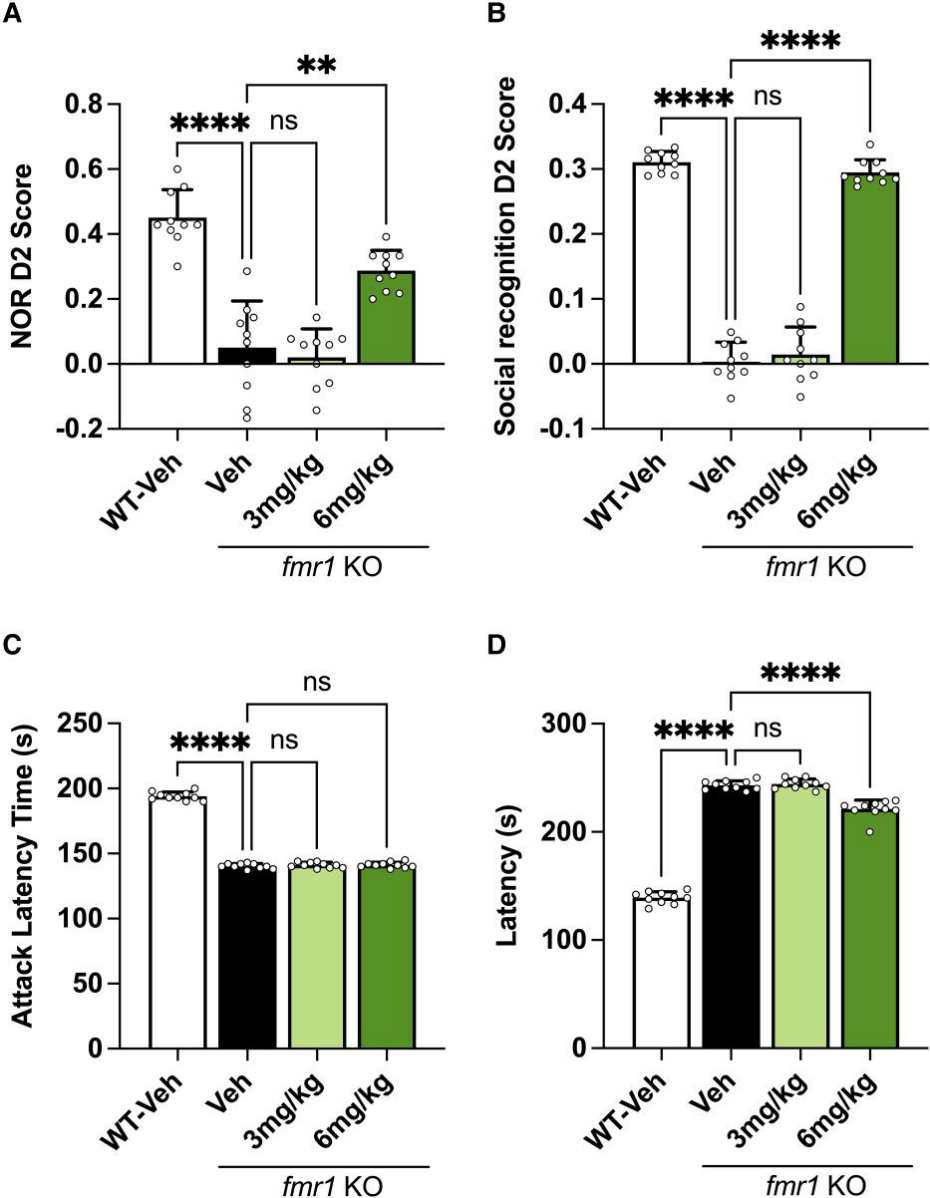
**Effects of chronic ibudilast dosing in *Fmr1* KO mice.** (**A**) NOR D2 score obtained by *Fmr1* KO and WT mice. Data analysed by Brown–Forsythe and Welch ANOVA [*F*(3,25.49) = 42.06, *P* < 0.0001] followed by Dunnett’s T3 multiple comparison test. (**B**) SR D2 score obtained by *Fmr1* KO and WT mice. Data analysed by Brown–Forsythe and Welch ANOVA [*F*(3,23.71) = 344.8, *P* < 0.0001] followed by Dunnett’s T3 multiple comparison test. (**C**) Attack latency time measured in seconds (s). Data analysed by Brown–Forsythe and Welch ANOVA [*F*(3,29.52) = 1195, *P* < 0.0001] followed by Dunnett’s T3 multiple comparison test. **(D)** Latency in seconds (s) to eat novel food for WT and *Fmr1* KO mice. Data analysed by Brown–Forsythe and Welch ANOVA [*F*(3,27.18) = 719.5, *P* < 0.0001] followed by Dunnett’s T3 multiple comparison test. Bars indicate mean values (mean ± SEM). Points correspond to values from individual mice. Asterisks represent significant change; ns, not significant; ****P < 0.0001 and **P = 0.0013 versus *Fmr1* KO vehicle group. *n* = 10.

As previously demonstrated, the *Fmr1* KO vehicle–treated mice are unable to perform in the NOR task due to a reduced cognitive capacity. Ibudilast treatment was however able to reverse this cognitive deficit dose dependently, with the 6 mg/kg group improving the D2 score in *Fmr1* KO mice. The 3 mg/kg ibudilast–treated group was unable to reverse the cognitive deficit in the NOR assay, giving a comparable D2 score to that of the *Fmr1* KO vehicle–treated group (Fig. 3A).

The partition test is a measure of social recognition (SR) that works on the same principle as NOR, except in this instance the test animals need to differentiate between a novel and a familiar mouse. The *Fmr1* KO vehicle–treated mice showed no preference for either the novel or the familiar mouse, as demonstrated by their low D2 score. The 6 mg/kg ibudilast–treated group was able to reverse the SR deficit in *Fmr1* KO mice back to comparable levels observed for the WT vehicle group. The 3 mg/kg-treated group showed no efficacy in *Fmr1* KO mice in the SR test (Fig. 3B).

Attack latency measures the time it takes for a mouse to attack a resident intruder and is a measure of aggression. The *Fmr1* KO vehicle–treated group displays an aggressive phenotype as demonstrated by the significantly reduced latency to attack an intruder mouse. Neither the 3 nor the 6 mg/kg ibudilast–dosed groups were able to reduce this aggressive phenotype (Fig. 3C). Hyponeophagia is a measure of anxiety by introducing novel food in a novel environment. The *Fmr1* KO vehicle–treated mice experience an increased latency to eat novel food compared to the WT vehicle–treated group. The 3 mg/kg ibudilast dose was not efficacious for reducing hyponeophagia; however, the 6 mg/kg dose showed a subtle but significant decrease in latency (Fig. 3D).

The 6 mg/kg ibudilast dose showed limited efficacy for normalizing behaviours such as aggression and hyponeophagia; however, this dose was effective for improving cognition in *Fmr1* KO mice as assessed by both NOR and the SR test. In contrast to this, gaboxadol was highly efficacious for normalizing several behaviours but showed no efficacy for improving cognition in *Fmr1* KO mice. On this basis, it was decided to test the efficacy of gaboxadol and ibudilast in combination and to assess whether the co-treatment of these drugs could improve the cognitive deficits as well as the behavioural phenotypes in *Fmr1* KO mice.

### Ibudilast and gaboxadol improve behavioural phenotypes of *Fmr1* KO mice

The efficacious doses of gaboxadol, 0.5 and 1.5 mg/kg, along with the efficacious ibudilast dose, 6 mg/kg, were selected to test the efficacy of combination treatments. To further explore the ibudilast 6 mg/kg dose, it was decided to compare once daily dosing (QD) with twice daily dosing (BID). Table 2 outlines the study design for comparing ibudilast or gaboxadol monotherapy to their combination. All drugs were dosed for 14 days prior to any behaviour testing, and dosing continued until the conclusion of all behaviour work. These animals were then subjected to a 14-day washout period during which the drug treatment was withdrawn and behavioural assessments (open field and self-grooming) were performed 14 days after the last dose of drug or vehicle to monitor the potential for sustained disease modification.

**Figure 4:**
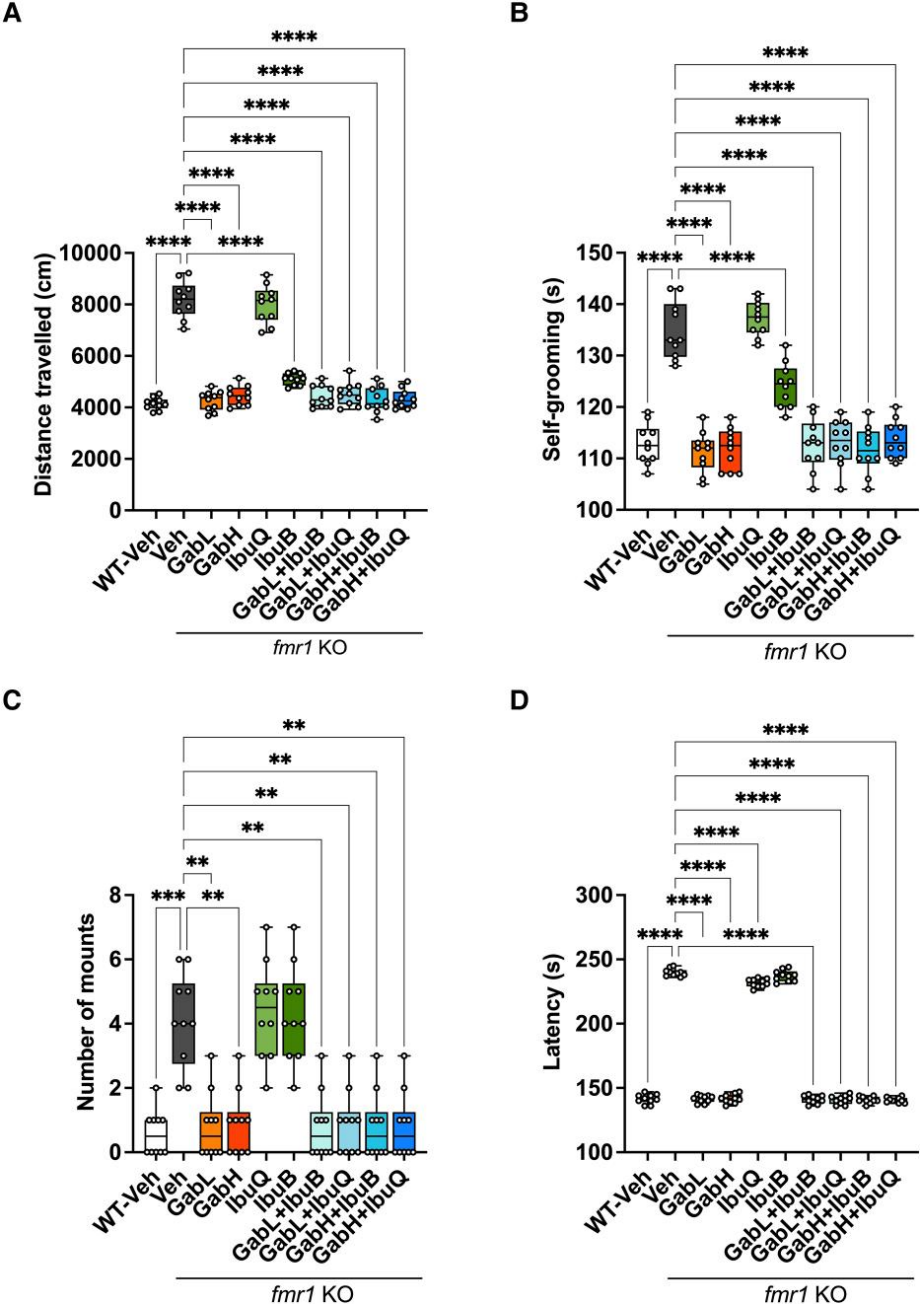
**Chronic ibudilast and gaboxadol treatment improves behavioural phenotypes in *Fmr1* KO mice.** (**A**) The total distance travelled (cm) by *Fmr1* KO and WT mice in the open field over 30 min. Data analysed by Brown–Forsythe and Welch ANOVA [*F*(9,58.09) = 111.3, *P* < 0.0001] followed by Dunnett’s T3 multiple comparison test. (**B**) Time in seconds (s) *Fmr1* KO and WT mice spent self-grooming. Data analysed by one-way ANOVA [*F*(9,90) = 53.33, *P* < 0.0001] followed by Dunnett’s multiple comparison test. (**C**) Aggression measured by the number of mounts performed by *Fmr1* KO and WT mice. Data analysed by the Kruskal–Wallis test (*P* < 0.0001) followed by Dunn’s multiple comparison test. (**D**) Latency, in seconds (s), for *Fmr1* KO and WT mice to eat novel food. Data analysed by one-way ANOVA [*F*(9,90) = 2068, *P* < 0.0001] followed by Dunnett’s multiple comparison test. Bars indicate mean values (mean ± SEM). Points correspond to values from individual mice. Asterisks represent significant change: ****P < 0.0001, ***P = 0.0008, and **P < 0.005 versus *Fmr1* KO vehicle group. *n* = 10. GabL, gaboxadol 0.5 mg/kg; GabH, gaboxadol 1.5 mg/kg; IbuB, ibudilast 6 mg/kg BID; IbuQ, ibudilast 6 mg/kg QD.

Monotherapy gaboxadol, at both 0.5 and 1.5 mg/kg, was highly efficacious in reversing a number of behaviours typically associated with FXS (Fig. 4A–D). Monotherapy gaboxadol, 0.5 and 1.5 mg/kg, significantly normalized the hyperactivity in *Fmr1* KO mice to levels observed in WT vehicle–treated mice (Fig. 4A).

Monotherapy ibudilast, 6 mg/kg BID, also significantly reduced hyperactivity in *Fmr1* KO mice, however not to levels observed for the WT vehicle–treated mice. The 6 mg/kg QD ibudilast dose showed no efficacy in the open field, inferring that twice daily dosing of ibudilast is more efficacious than once daily dosing for this behaviour test. From these data, we can conclude that gaboxadol was more effective in reducing hyperactivity in the *Fmr1* KO mouse than BID ibudilast (Fig. 4A).

All the combination treatments of gaboxadol (0.5 or 1.5 mg/kg) with ibudilast (QD or BID) were highly effective at reducing hyperactivity in *Fmr1* KO mice back to levels observed in WT vehicle–treated mice. Despite ibudilast BID monotherapy showing partial efficacy and ibudilast QD monotherapy demonstrating no efficacy, in the open field, combining these doses with either 0.5 or 1.5 mg/kg gaboxadol significantly reverted the hyperactivity in *Fmr1* KO mice to WT levels (Fig. 4A).

Repetitive self-grooming was used as a measurement of stereotypy. Both monotherapy doses of gaboxadol significantly reduced the repetitive behaviour in *Fmr1* KO mice to levels observed in WT vehicle–treated mice (Fig. 4B). Ibudilast BID also significantly reduced self-grooming in the *Fmr1* KO mice, however not levels observed in the WT group (*P* < 0.001 versus WT vehicle). Ibudilast QD did not demonstrate efficacy for stereotypy in *Fmr1* KO mice. Despite this, all combinations of gaboxadol (0.5 or 1.5 mg/kg) and ibudilast (QD or BID) were able to fully revert the repetitive grooming phenotype in *Fmr1* KO mice to WT vehicle levels (Fig. 4B).

Gaboxadol monotherapy (0.5 or 1.5 mg/kg) was able to significantly reduce the aggressive phenotype in *Fmr1* KO mice to levels observed in the WT group (Fig. 4C). Neither QD nor BID ibudilast showed any efficacy at ameliorating the aggression in *Fmr1* KO mice. Despite this, all combinations of gaboxadol (0.5 or 1.5 mg/kg) and ibudilast (QD or BID) significantly reduced the aggressive phenotype in *Fmr1* KO mice to levels observed in the WT group (Fig. 4C).

Monotherapy gaboxadol treatment (0.5 or 1.5 mg/kg) significantly reduced anxiety, as measured by hyponeophagia, in *Fmr1* KO mice to levels observed in WT mice (Fig. 4D). Ibudilast QD displayed a subtle but significant amelioration of anxiety in *Fmr1* KO mice; however, this reduction was not to levels observed for WT mice (*P* < 0.0001 versus WT vehicle). Ibudilast BID showed no efficacy for reducing anxiety in *Fmr1* KO mice. All combinations of gaboxadol (0.5 or 1.5 mg/kg) and ibudilast (BID or QD) significantly rescued the anxious phenotype in *Fmr1* KO mice to levels observed in the WT group. Importantly, no negative interactions, in terms of efficacy, were observed with any ibudilast–gaboxadol combination treatments for the behaviours measured.

**Figure 5:**
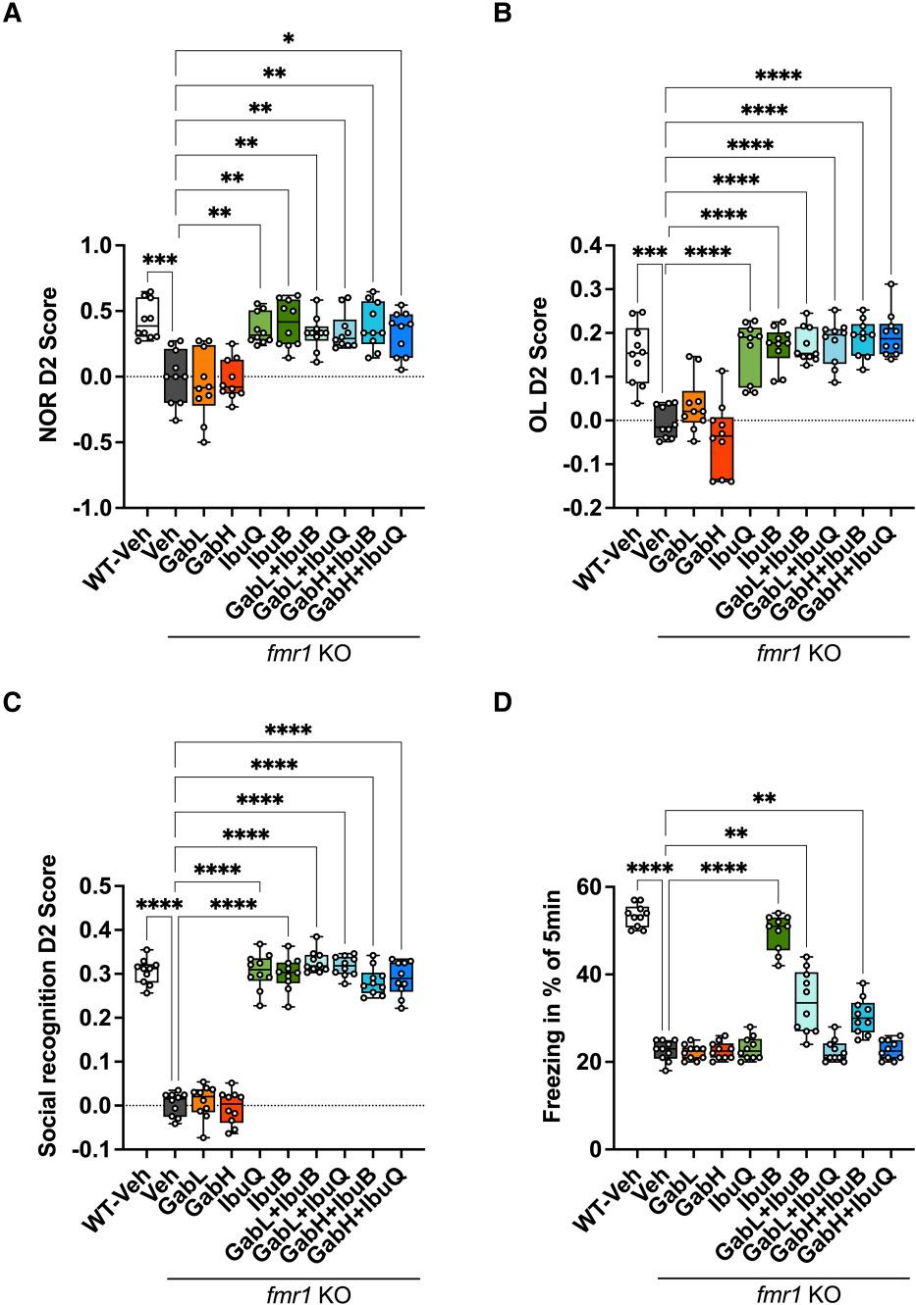
**Ibudilast and ibudilast–gaboxadol combinations reverse cognitive deficits in *Fmr1* KO mice.** (**A**) NOR D2 score obtained by *Fmr1* KO and WT mice. Data analysed by Brown–Forsythe and Welch ANOVA [*F*(9,70.70) = 12.73, *P* < 0.0001] followed by Dunnett’s T3 multiple comparison test. (**B**) OL D2 score obtained by *Fmr1* KO and WT mice. Data analysed by Brown–Forsythe and Welch ANOVA [*F*(9,71.23) = 24.37, *P* < 0.0001] followed by Dunnett’s T3 multiple comparison test. (**C**) SR D2 score obtained by *Fmr1* KO and WT mice. Data analysed by one-way ANOVA [*F*(9,90) = 189.9, *P* < 0.0001] followed by Dunnett’s multiple comparison test. (**D**) Percent time *Fmr1* KO and WT mice were immobile (freezing) over 5 min. Data analysed by Brown–Forsythe and Welch ANOVA [*F*(9,39.34) = 115.5, *P* < 0.0001] followed by Dunnett’s T3 multiple comparison test. Bars indicate mean values (mean ± SEM). Points correspond to values from individual mice. Asterisks represent significant change: ****P < 0.0001, ***P < 0.0005, **P < 0.001, and *P = 0.018 versus *Fmr1* KO vehicle group. *n* = 10. GabL, gaboxadol 0.5 mg/kg; GabH, gaboxadol 1.5 mg/kg; IbuB, ibudilast 6 mg/kg BID; IbuQ, ibudilast 6 mg/kg QD.

Cognitive integrity was assessed using four separate cognitive assays: NOR (Fig. 5A), OL (Fig. 5B), SR (Fig. 5C) and cFC (Fig. 5D). Each of these cognitive tasks utilizes bespoke brain regions and connections, with some degree of overlap. The *Fmr1* KO vehicle–treated mice were significantly impaired in all the cognitive assays in comparison to WT mice. Monotherapy gaboxadol treatment was unable to improve the performance of the *Fmr1* KO mice in any of the cognitive tasks (Fig. 5A–D). Contrary to this, monotherapy ibudilast (BID or QD) was able to completely reverse the cognitive deficit in *Fmr1* KO mouse as measured in the NOL, OL and SR assays, back to levels observed in the WT animals (Fig. 5A–C). This efficacy in cognition was maintained when ibudilast (QD or BID) was combined with gaboxadol (0.5 or 1.5 mg/kg). Ibudilast BID was also able to reverse the cognitive deficit in *Fmr1* KO mice observed in the cFC task back to levels observed in the WT mice. Ibudilast QD treatment was not efficacious in the cFC task, and this lack of efficacy was maintained when combined with gaboxadol (Fig. 5D). Surprisingly, the potency observed for ibudilast BID monotherapy was significantly reduced when combined with gaboxadol 0.5 mg/kg (*P* < 0.01 versus ibudilast BID) or 1.5 mg/kg (*P* < 0.0001 versus ibudilast BID) for the cFC task (Fig. 5D). These combinations, however, still showed a significant improvement in cFC-associated learning and memory in comparison to the *Fmr1* KO vehicle–treated mice (*P* = 0.0058 for ibudilast BID + 0.5 mg/kg or *P* = 0.001 for ibudilast BID + 1.5 mg/kg).

### Ibudilast and gaboxadol maintain efficacy for *Fmr1* KO behavioural phenotypes following washout

Mice that followed the dosing regimen outlined in Table 2 were subjected to a 14-day washout period during which time the animals received no drug. Following the 14-day washout period, mice were subjected to open-field and self-grooming assessment.

**Figure 6:**
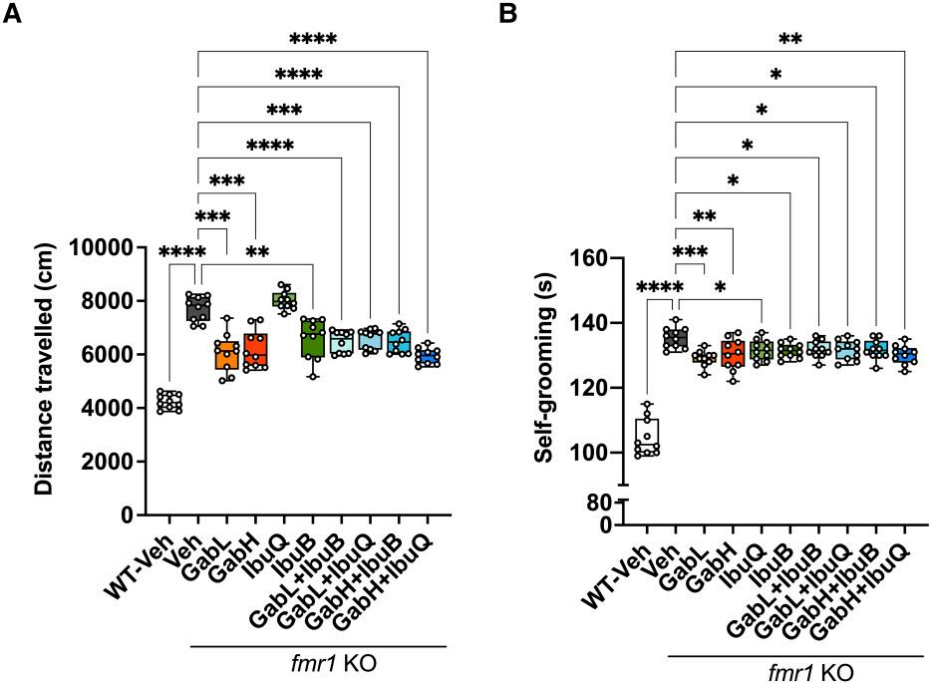
**Effects of 14-day washout of ibudilast and gaboxadol on hyperactivity and stereotypy in *Fmr1* KO mice.** (**A**) The total distance travelled (cm) in the open field over 30 min for *Fmr1* KO and WT mice. Data analysed by Brown–Forsythe and Welch ANOVA [*F*(9,60.15) = 41.593, *P* < 0.0001] followed by Dunnett’s T3 multiple comparison test. (**B**) Time in seconds (s) *Fmr1* KO and WT mice spent self-grooming. Data analysed by one-way ANOVA [*F*(9,90) = 61.01, *P* < 0.0001] followed by uncorrected Fisher’s least significant difference (LSD). Data also analysed by Fisher’s LSD without adjustment for multiple comparisons. Bars indicate mean values (mean ± SEM). Points correspond to values from individual mice. Asterisks represent significant change: ****P < 0.0001, ***P < 0.0005, **P < 0.01, and *P < 0.05 versus *Fmr1* KO vehicle group. *n* = 10. GabL, gaboxadol 0.5 mg/kg; GabH, gaboxadol 1.5 mg/kg; IbuB, ibudilast 6 mg/kg BID; IbuQ, ibudilast 6 mg/kg QD.

Following drug washout, all treatments, monotherapy and combinations, maintained their efficacy for significantly reducing hyperactivity and stereotypy in *Fmr1* KO mice, although with reduced potency (Fig. 6A and B).

## Discussion

We present here the combination of ibudilast and gaboxadol for the treatment of FXS. By simultaneously targeting pathways, which are dysregulated in FXS, we are able to rescue more phenotypes than can be achieved with a monotherapy treatment. Altered cAMP metabolism and reduced inhibitory GABA modulation have been proposed to be key pathophysiological pathways in FXS with each contributing to different, and sometimes overlapping, symptoms in the clinical population.

Much of ibudilast’s efficacy is targeted towards reversing the cognitive deficits in *Fmr1* KO mice, particularly for the BID group where cognition was improved for all the cognitive assays tested: NOR, OL, SR and cFC. We can speculate that the beneficial effects of ibudilast span across the brain as each of these cognitive assays targets different brain regions and connections. As confirmation, PDE4D inhibition has already been shown to improve cognition in this patient population.^15^

Ibudilast does potentially have advantages over selective PDE4 inhibitors due to its broad selectivity profile against PDE3, PDE4, PDE10 and PDE11.^11^ Despite this broad selectivity profile, ibudilast is more selective for PDE4 and PDE10, with IC_50_ values in the lower micromolar range, compared to PDE3 and PDE11 where IC_50_ values are in the 10 μM range.^11^ This implies that ibudilast’s PDE efficacy is likely coming from its inhibition of PDE4 and PDE10. Reduced cAMP levels in FXS patient cells were first identified by Berry-Kravis *et al.*^16^ decades ago. Although PDE4 is a minor target of FMRP, several preclinical and clinical studies have supported PDE4 inhibition as a viable target in FXS. The initial findings supporting PDE4 as a therapeutic target in FXS come from work in *Drosophila* and later mouse^40-42^ and most recently a Phase 2 clinical trial.^15^ PDE10 is however a target of FMRP^43^ and as a result levels are elevated in FXS. PDE10 is highly expressed in medium spiny neurons (MSNs) of the striatum^44,45^ that modulates the input and processing of cortical information by the basal ganglia circuit.^46,47^ The basal ganglia is responsible for motor control, motor learning, executive functions, behaviours and emotions^48^ and is dysregulated in FXS^49-51^ leading to impaired executive function skills such as working memory, attention and inhibitory control.^52^ Beneficial effects of PDE10 inhibition have been demonstrated in *Fmr1* KO mice by normalizing EEG recorded chirp ITPC.^53^ This suggests that PDE10 inhibition reduces auditory hypersensitivity, a debilitating condition that can lead to language delays, social anxiety and stereotypy^54^ all common symptoms in FXS patients. Ibudilast has the potential to improve auditory hypersensitivity and reverse neural deficits within the basal ganglia through PDE10 modulation. Similarly, PDE2, another FMRP target, has also shown promise as a potential therapeutic target in a mouse model of FXS.^55^ Ibudilast is however not selective against PDE2.

PDE10 modulates cAMP and cGMP, both of which are essential for axonal, neurite and dendritic growth, maintenance and maturation.^56^ The importance of these cyclic nucleotides was demonstrated when cAMP improved dendritic spine morphology in a mouse model of FXS.^42^ Dense immature neuronal spines are a hallmark of FXS that contributes to cognitive impairment. Another factor affecting cognition is reduced levels of glutamate in the hippocampus^57^ and cortex^58^ of FXS mouse models. However, glutamate levels could be restored by increasing cGMP levels, which promotes presynaptic glutamate release.^59^ PDE inhibition may also protect against overactive mGluR5 signalling, another hallmark of FXS, which leads to long-term depression and eventual synapse loss.^37^ Activation of protein kinase A (PKA), by cAMP, directly binds to, phosphorylates and inhibits both isoforms of glycogen synthase kinase (GSK3), which is located downstream of the mGluR5 signalling pathway.^60^ Inhibition of GSK has been shown to reverse the effects of overactive mGluR5 signalling and improve cognition in FXS mouse models.^37^

Ibudilast also has potent anti-inflammatory properties, demonstrated by its ability to reduce proinflammatory cytokines and reactive oxygen species (ROS) in microglial cells.^61,62^ In addition to this, ibudilast is able to bind and inhibit toll-like receptor 4 (TLR4).^63^ The astrocyte-secreted factor tenascin C (TNC), which is an endogenous ligand of TLR4, has been found to be elevated in FXS astrocytes leading to increased extracellular interleukin-6 (IL-6) levels.^64^ Elevated IL-6 increases excitatory synapse formation while impairing the development of inhibitory synapses.^65,66^ This disruption in excitatory inhibitory imbalance is a key feature in FXS leading to changes in network synchrony.^67^ Ibudilast could reduce IL-6 levels by inhibiting TNC-dependent TLR4 activation. Ibudilast is also able to protect against ROS,^68^ which could prove favourable for FXS, as preclinical models have elevated lipid peroxidation and protein oxidation in the brain, caused by ROS. Elevated ROS, in FXS, are a result of increased activity of the nicotinamide adenine dinucleotide phosphate (NADPH) oxidase and deficits in the ROS-scavenging glutathione system.^69^

Contrary to ibudilast, gaboxadol was unable to improve cognition in *Fmr1* KO mice for any of the cognitive assays tested. We did demonstrate that gaboxadol effectively normalized behaviours such as hyperactivity, aggression, stereotypy and anxiety in *Fmr1* KO mice. Many of these behaviours are exhibited by FXS patients and may be a result of decreased GABAergic function,^19^ which is an excitatory/inhibitory imbalance brought about by reduced GABA_A_ receptor availability, alterations in GABA transport,^21^ synthesis and release.^20,22,23^ Several ion channels are also differentially expressed in FXS, which contributes to the excitatory/inhibitory imbalance leading to altered neuronal resting membrane potential that ultimately affects neuronal development, network connections and interactions.^7^ Two independent groups have demonstrated gaboxadol’s efficacy in FXS.^23,32,33^ Each of these groups used different KO mouse models, *Fmr1* KO1 and *Fmr1* KO2, bred on different background strains, which added to the complexity and heterogeneity of the validation. Gaboxadol has also recently demonstrated efficacy in a Phase 2a clinical trial in FXS patients based on clinician- and caregiver-rated end-points, which assessed behaviours such as hyperactivity, irritability, stereotypy and anxiety.^24^ The improvements for these clinical end-points mirror our findings, for gaboxadol monotherapy, as well as previously published work.^33^

**Figure 7:**
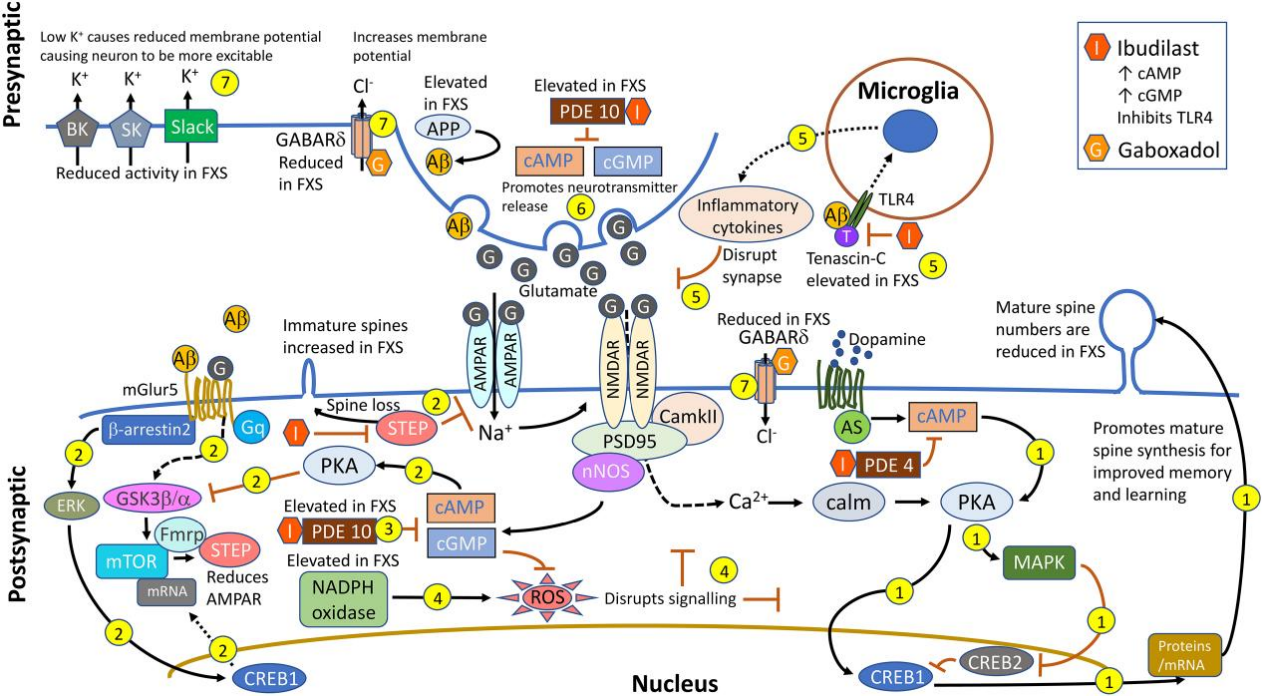
**Proposed MoA for ibudilast and gaboxadol in FXS.** (1) cAMP facilitates learning and long-term memory. Ibudilast increases cAMP and cGMP levels through PDE4 and PDE10 inhibition. (2) Ibudilast may decrease mGluR5 signalling by cAMP-dependent PKA inhibition of GSK3. (3) PDE10 is elevated in FXS, and ibudilast inhibits PDE10 that improves executive function and hypersensitivity. (4) Ibudilast protects against elevated ROS. NADPH oxidase is elevated in FXS causing an increase in ROS. (5) Ibudilast inhibits TLR4. TNC is elevated in FXS and promotes the secretion of proinflammatory cytokines through TLR4 activation. (6) Glutamate levels are reduced in FXS, and cAMP and cGMP promote neurotransmitter release. (7) Gaboxadol promotes delta subunit signalling. GABA_A_ receptor delta subunit levels are reduced in FXS leading to behaviour abnormalities. This figure represents biological pathways that are potentially rescued by ibudilast and gaboxadol treatment and does not represent all biological processes disrupted as a result of FMRP loss.

Gaboxadol is highly selective against the extrasynaptic GABA_A_ receptor delta subunit, levels of which are significantly reduced in FXS.^70,71^ Dysregulated expression of the delta subunit can impact behaviour, as was observed when it was selectively knocked out of cerebral granule cells of WT mice. The KO mice were hyperactive, displayed stress related behaviours, were anxious and were socially withdrawn.^72^ These mice display a phenotype not dissimilar to that observed for FXS mouse models, which highlights the influence the delta subunit has on behavioural outcomes. In addition, a gene dosage effect was evident with homozygous KO mice displaying a greater phenotype than heterozygous KO mice. Importantly, certain behaviours could be reversed in heterozygous KO mice following gaboxadol treatment,^72^ which gives some rationale for targeting this subunit in FXS and potentially other neurodevelopmental disorders (NDDs). The proposed therapeutic effects gaboxadol and ibudilast may have on FXS pathophysiology are illustrated in Fig. 7.

Our results presented here demonstrate that by pharmacologically targeting two independent pathophysiological pathways in FXS, using two drugs with different mechanisms of action (MoAs), specific phenotypes can be treated. We also demonstrate that when these two drugs are administered as a combination treatment, we were able to rescue both the cognitive deficits and the behaviour abnormalities in *Fmr1* KO mice. Each combinatorial treatment significantly improved hyperactivity, stereotypy and hyponeophagia. The combination treatments were also able to significantly reduce aggression and improve cognition in *Fmr1* KO mice as assessed by the NOR, OL and SR assays. Only the combination treatments of gaboxadol at 0.5 mg/kg and ibudilast at 6 mg/kg BID and gaboxadol at 1.5 mg/kg and ibudilast at 6 mg/kg BID significantly improved spatially related contextual memory in the cFC assay, although with a reduced response or potency in comparison to monotherapy ibudilast 6 mg/kg BID treatment. This reduced potency is thought to be the result of the anxiolytic effects of gaboxadol, which is able to reduce hyperexcitability in the amygdala of a FXS mouse model.^23^ In addition, it has been demonstrated that WT mice dosed with gaboxadol show a similar reduced response in the fear conditioning assay in comparison to vehicle controls due to the anxiolytic effect of gaboxadol.^32^

Importantly, both monotherapy and combination treatments maintained their efficacy following chronic dosing and showed no signs of adverse effects or pharmacoresistance. In addition, these drugs maintained efficacy, although with some reduced potency, following a 14-day washout that indicates a degree of disease modification. Both ibudilast and gaboxadol have been shown to be safe and well tolerated in patients, in particular gaboxadol that showed a good safety profile in FXS patients.^24^ In addition, the combination of ibudilast and gaboxadol was well tolerated in the mouse. Animals receiving the combination were able to perform the behaviour assays to the same level as WT vehicle animals, showing they were not distressed as a result of the combination treatment. However, tolerability of the combination will need to be confirmed in a second species and/or in an appropriately phased clinical study. In summary, this polypharmacological approach of targeting different pathophysiological pathways allows for a larger number of symptoms to be addressed in this heterogeneous patient population and could revolutionize drug discovery going forward, particularly for other NDDs.

## Supplementary material

Supplementary material is available at *Brain Communications* online.

## Supplementary Material

fcad353_Supplementary_Data

## Data Availability

The data supporting the results of this study are available upon reasonable request to the corresponding author.
