# Supplementary material for: A novel combination treatment for fragile X syndrome predicted using computational methods
Source: Brain Commun. 2024 Jan 15;6(1):fcad353. doi: 10.1093/braincomms/fcad353 (PMC10789243; doi:10.1093/braincomms/fcad353)
Supplement: fcad353_Supplementary_Data [file fcad353_supplementary_data.docx]

**
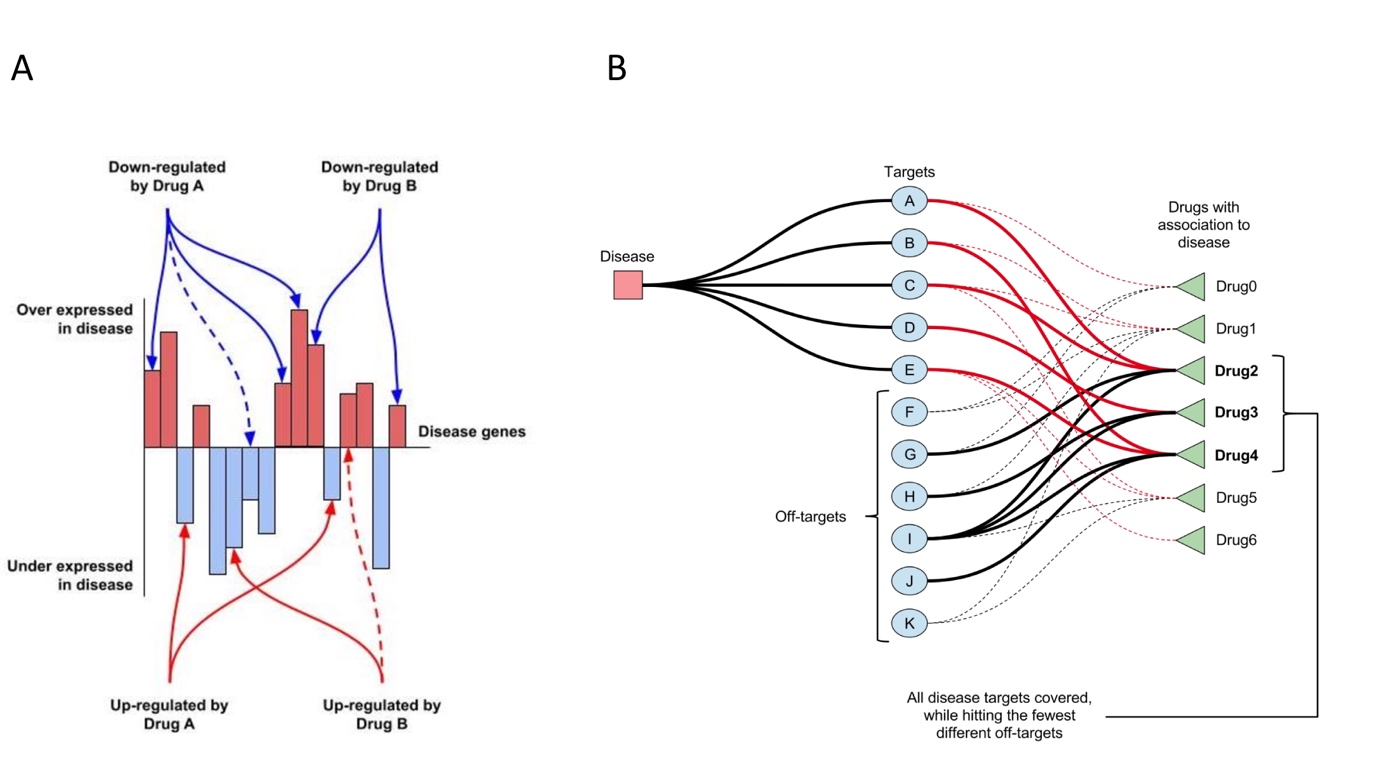
**

**Supplementary Figure 1 Schematic depicting the approach taken by CGEM and TargOpt.** (A) Schematic depicting the approach taken by CGEM which considers which genes in the disease are over- and under-expressed, and finds combinations of drugs that may maximize the reversal of this mis-regulation of differentially expressed disease genes. (B) Schematic depicting the approach taken by TargOpt, which finds combinations of drugs that maximize coverage of relevant disease on-targets while minimizing the coverage of off-targets for a set of disease targets.

**
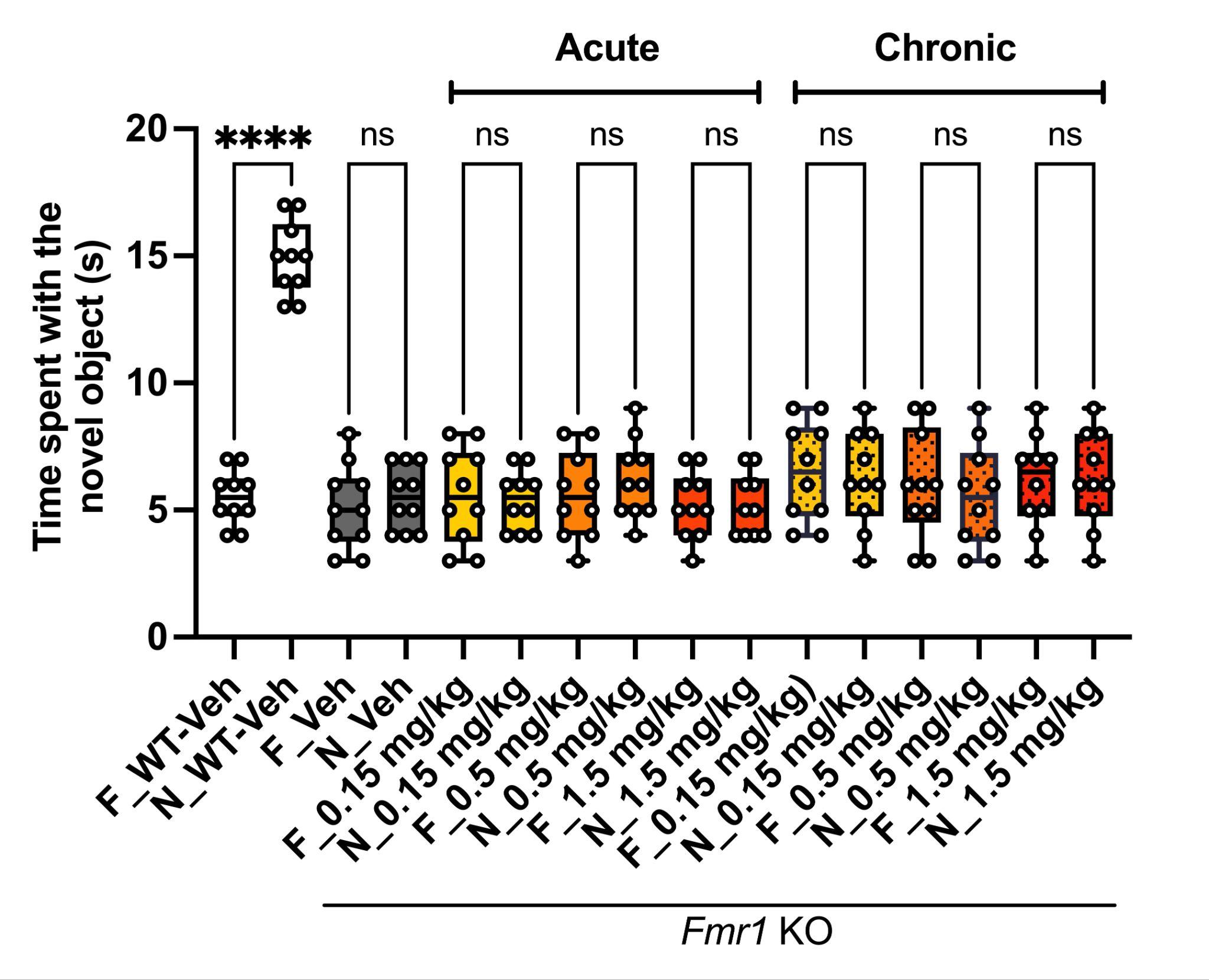
**

**Supplementary Figure 2 NOR time spent investigating novel (N) and familiar (F) objects.** Data analysed by ordinary one-way ANOVA (*F*(15, 144) = 19.62, *P* < 0.0001) followed by Sidak’s multiple comparisons test. Bars indicate mean values (mean ± SEM). Points correspond to values from individual mice. Asterix (*) represent significant change, **** *P* < 0.0001. *n* = 10. GabL: Gaboxadol 0.5mg/kg, GabH: Gaboxadol 1.5mg/kg.

**
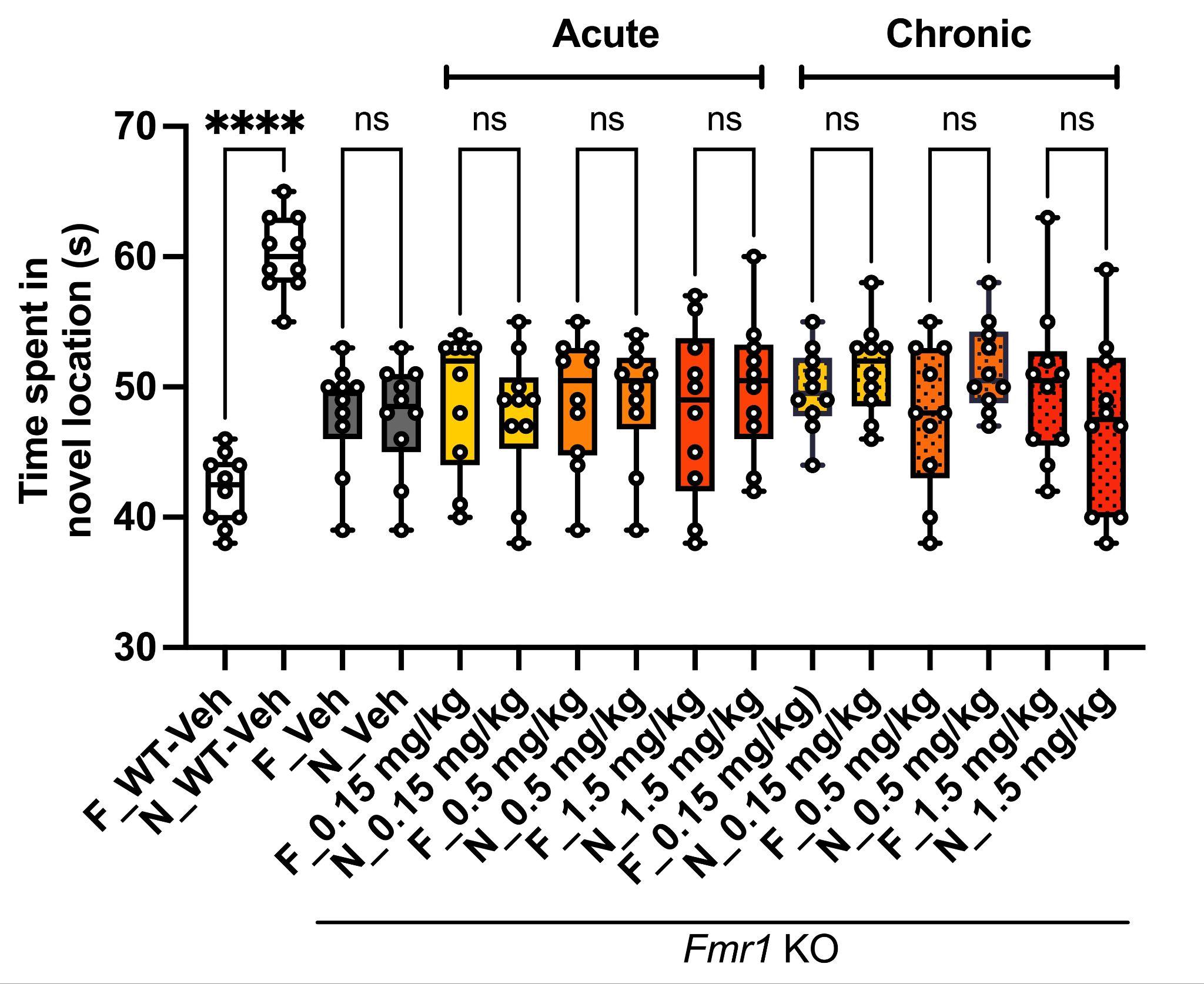
Supplementary Figure 3 OL time spent investigating novel (N) and familiar (F) locations.** Data analysed by ordinary one-way ANOVA (*F*(15, 144) = 5.601, *P* < 0.0001) followed by Sidak’s multiple comparisons test. Bars indicate mean values (mean ± SEM). Points correspond to values from individual mice. Asterix (*) represent significant change, **** *P* < 0.0001. *n* = 10. GabL: Gaboxadol 0.5mg/kg, GabH: Gaboxadol 1.5mg/kg.
